# A Potential Linking between Vitamin D and Adipose Metabolic Disorders

**DOI:** 10.1155/2020/2656321

**Published:** 2020-02-18

**Authors:** Zhiguo Miao, Shan Wang, Yimin Wang, Liping Guo, Jinzhou Zhang, Yang Liu, Qiyuan Yang

**Affiliations:** ^1^College of Animal Science and Veterinary Medicine, Henan Institute of Science and Technology, Xinxiang, Henan 453003, China; ^2^Department of Molecular, Cell and Cancer Biology, University of Massachusetts Medical School, Worcester, MA 01605, USA

## Abstract

Vitamin D has been discovered centuries ago, and current studies have focused on the biological effects of vitamin D on adipogenesis. Besides its role in calcium homeostasis and energy metabolism, vitamin D is also involved in the regulation of development and process of metabolic disorders. Adipose tissue is a major storage depot of vitamin D. This review summarized studies on the relationship between vitamin D and adipogenesis and furthermore focuses on adipose metabolic disorders. We reviewed the biological roles and functionalities of vitamin D, the correlation between vitamin D and adipose tissue, the effect of vitamin D on adipogenesis, and adipose metabolic diseases. Vitamin D is associated with adipogenesis, and vitamin D supplements can reduce the burden caused by metabolic diseases. The review provides new insights and basis for medical therapy on adipose metabolic diseases.

## 1. Introduction

Vitamin D is an essential nutrient for the prevention of rickets and is responsible for the intestinal absorption of calcium, phosphate, and magnesium [1, 2]. Vitamin D can be obtained from food, but most of it is synthesized from 7-dehydrocholesterol in the skin via ultraviolet irradiation [3, 4]. The mechanism of vitamin D action is through its active form, 1*α*, 25-dihydroxyvitamin D_3_ [1*α*, 25(OH)_2_D_3_], which regulates the transcription of target genes and thus plays an important role in calcium homeostasis and metabolism [5–7]. Vitamin D deficiency or insufficiency is still a common issue in developing countries [8, 9]. Among 734 adolescents ranging from 12 to 18 years, 87.6% of participants had vitamin D deficiency [10]. Aside from its involvement in calcium and bone mineralization, vitamin D has multiple functions in adipose tissue, adipogenesis, glucose-insulin homeostasis, cell growth, and so on [11–13].

Adipose tissue is a vital organ in energy homeostasis and glucose metabolism [14–16]. Adipose tissue is also an endocrine organ secreting proteins and releasing fatty acids [17, 18]. It is composed of various cell types, including mature adipocytes, preadipocytes, fibroblasts, macrophages, and immune cells. The predominant cell types existing in adipose tissue are mature adipocytes. Preadipocytes differentiate into mature adipocytes in adipose tissue, and this process requires the regulation of transcription factors (peroxisome proliferator-activated receptor (PPAR), CCAAT enhancer-binding protein (C/EBP), and Kruppel-like factor proteins) [19, 20]. Vitamin D is mainly stored in adipose tissue, while vitamin D receptor (VDR) is expressed in adipose tissue [21, 22]. VDR is an activated transcription factor of active vitamin D. Previous studies investigated the effect of vitamin D on adipogenesis in animal models [23, 24], and results suggested that vitamin D exerts antiadipogenic activity in 3T3-L1 preadipocytes [25–27]. Meanwhile, vitamin D deficiency or insufficiency is involved in the regulation of insulin secretion, glucose levels, and inflammation causing adipose metabolic diseases, such as obesity, multiple sclerosis, diabetes, and fatty liver [28–32].

Hence, we reviewed the correlation between vitamin D and adipose tissue, along with related metabolic disorders (diabetes, nonalcohol fatty liver, and cardiovascular diseases). This study aimed to establish the relationship between vitamin D and metabolic disorders and furthermore to determine whether such disorders are affected by vitamin D supplementation, adipose vitamin D metabolism, and increased or reduced vitamin D activation. The vitamin D status, regulation of vitamin D by adipose tissue, effect of vitamin D on adipogenesis, and related metabolic diseases were also discussed.

## 2. Bioactivation of Vitamin D

**Figure 1:**
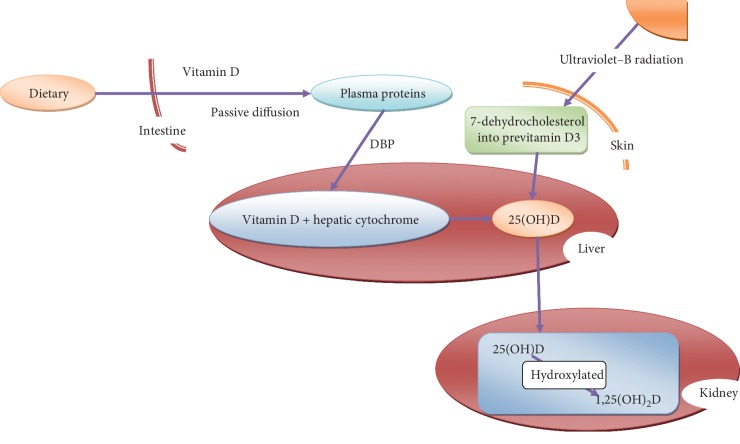
Bioactivation of vitamin D.

Only a small amount of fat-soluble vitamin D can be obtained from the diet and from supplements [33]. The major source of vitamin D is produced in the skin through a sunlight-dependent chemical reaction [4, 34]. Exposure of the skin to ultraviolet-B radiation from the sun converts 7-dehydrocholesterol into previtamin D_3_, which can be isomerized to vitamin D_3_ [35]. Vitamin D_3_ is then converted into calcifediol (25-(OH)D) in the liver and further hydroxylated into 1,25(OH)_2_D in the kidney (Figure 1) [36]. Normally, the serum concentration of 25-(OH)D is measured to determine an individual's vitamin D status in serum, whereas 1,25(OH)_2_D is the biological active form of vitamin D [37–39].

The biological activity of 1,25(OH)_2_D is mediated through binding to VDR [40]. VDR is also well-documented as calcitriol receptor and is a member of the steroid hormone nuclear receptor family [41]. In humans, VDR is encoded by the VDR gene [42]. VDR widely exists in tissues and cells, such as skeleton, kidney, renal, skin, and immunocytes [4]. In nuclear, 1*α*,25-(OH)_2_D_3_, which is the active form of vitamin D, is capable of binding to VDR and form a heterodimer with retinoid X receptor (RXR); the complex binds to RNA polymerase and VDR interacting protein, resulting in the regulation of DNA transcription [43, 44]. Downstream targets of VDR are involved in calcium homeostasis, immune response, and cancer development [45]. Hindered VDR expression can impact diverse diseases, including cardiovascular disease, diabetes, tumors, tuberculosis, and multiple sclerosis [46].

After dietary intake, vitamin D needs to be absorbed by meal of fat through passive diffusion in the intestine. The absorbed vitamin D is transported to the liver by binding to diverse plasma proteins, such as vitamin D-binding protein (DBP), *β*-lipoprotein, and albumin [47–49]. DBP is an important carrier protein that can attenuate the toxicity of vitamin D by limiting its bound metabolites to target cells [50]. In the liver, vitamin D is converted into 25(OH)D catalyzed by the several hepatic cytochrome P-450s [51–53]. Of note, the metabolite is released into plasma and transported to the kidney, where it is converted into 1,25(OH)D, and is finally transported throughout the body (Figure 1) [54]. After synthesis, absorption, and transport, active vitamin D is distributed to hydrophobic parts of tissues [55]. Unlike other fat-soluble vitamin, vitamin D is not stored in the liver (except in some fish livers) [56]. Vitamin D is mostly stored in adipose tissue, and a large amount of vitamin D is combined with lipid, resulting in release and metabolic difficulties [57, 58].

## 3. Vitamin D and Lipid Metabolism

### 3.1. Regulation of Activation of Vitamin D by Adipose Tissue

Aside from the important roles of vitamin D in intestinal calcium, phosphate uptake, and bone mass regulation, it is also involved in other processes, including cell growth, immune functions, inflammation regulation, and neuromuscular functions [59–63]. Vitamin D could be regulated by hydroxylation that includes two-step enzymatic processes. Hydroxylation is performed by enzymes CYP27B1, CYP2J2, CYP27A1, and CYP3A4 in the liver and CYP27B1 in the kidney [64]. These enzymes are consecutively expressed in subcutaneous adipose tissue (SAT) and visceral adipose tissue [65, 66]. The CYP27B1 gene is expressed in the SAT of lean individuals and in 3T3-L1 preadipocytes, and it is regulated by calcitonin, hormones, calcium, and phosphorus [53]. Thus, the location of these enzymes in adipose tissue could implicate the production of active vitamin D.

**Figure 2:**
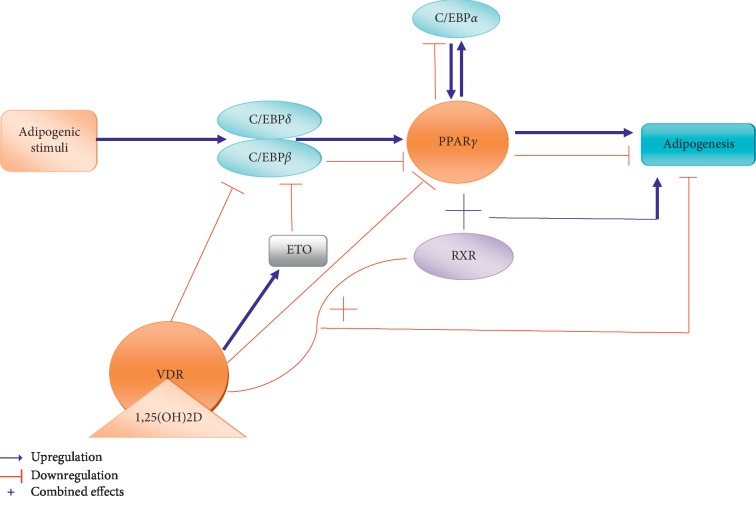
The relationship between vitamin D and adipogenesis. ETO: eight twenty-one. PPAR*γ*: peroxisome proliferator-activated receptor *γ*. C/EBP*α*: CCAAT enhancer binding protein *α*. C/EBP*β*: CCAAT enhancer binding protein *β*. C/EBP*δ*: CCAAT enhancer binding protein δ. VDR: vitamin D receptor. RXR: retinoid X receptor.

Previous reports showed that the concentrations of vitamin D in blood below 50 nmol/L indicated vitamin D deficiency, and the reference values of vitamin D levels in blood should exceed 75 nmol/L; lower serum vitamin D levels correlated with higher frequency of obesity and excessive body weight [67–69]. Circulating 25(OH)D level depends on the storage of vitamin D in adipose tissue, indicating that adipose tissue probably affects the activation, regulation, or action of obesity via regulation of vitamin D [57, 70, 71]. In the presence of calcitriol, adipogenesis is blocked by VDR via downregulating both C/EBP*β* nuclear protein levels and mRNA expression. In addition, 1,25(OH)2D3 allows for the upregulation of eight twenty-one (ETO), which is the core-repressor of C/EBP*β*, and finally leads to C/EBP*β* deficiency in adipogenesis [72]. VDR expression in 3T3-L1 cells inhabited PPAR*γ* mRNA levels, which decreased adipogenesis [73]. These data indicated that VDR reduced adipogenesis through decreasing the expression of C/EBP*β* and PPAR*γ* and increasing ETO expression. The molecular mechanism of inhibitory effect of VDR on adipogenesis maybe due to the fact that RXR is a heterodimeric partner for both PPAR*γ* and VDR, respectively, and that VDR leads to competition between RXR and PPAR*γ* to decrease adipogenesis (Figure 2). Previous studies confirmed the competitive relationship between VDR and PPAR*γ* for RXR [73–75]. Certainly, the mechanism underlying still needs to be proved by further investigation. Taken together, these investigations suggest that vitamin D plays a complex role through VDR and the transcription pathways in regulating adipogenesis.

### 3.2. Effect of Active Vitamin D on Adipogenesis

Adipogenesis is a cascade of differentiation that leads to adipocyte maturation. Adipocytes can affect many adipogenesis-related functions, such as adipokine secretion, lipid synthesis, fatty acid transfection, and insulin signaling response [76]. A vast amount of molecular interactions is involved during adipogenesis, and the main component is the expression of C/EBP*β* and PPAR*γ* [77]. C/EBP*β* and C/EBP*δ* are expressed in the early stage of adipogenesis. The adipogenesis is promoted under the regulation of C/EBP*α*, *β*, and *δ* [78]. 1,25(OH)2D3 can inhibit 3T3-L1 preadipocyte differentiation by downregulating C/EBP*β* and PPAR*γ* (Figure 2) [79, 80]. When combined with genistein, 1,25(OH)2D3 inhibits adipocyte lipid-binding protein 2 expression and fat accumulation in 3T3-L1 preadipocytes [81].

VDR plays a vital role in adipogenesis. The activity of vitamin D is performed through 1,25(OH)2D3-VDR actions, and the target organs of VDR are the liver, kidney, genitourinary tract, intestine, bone, brain, and various immune cells [5, 82]. VDR is expressed at the early stage of adipose differentiation [83]. Macrophage inflammation can induce the expression of VDR [84]. Knockdown of VDR in mice could lead to low-fat mass, high rates of *β*-oxidation, and adipogenesis inhibition; in VDR^+/+^ cells, 1,25(OH)2D3 treatment can block adipogenesis [72, 83]. In the absence of 1,25(OH)2D3, unliganded VDR also inhibits 3T3-L1 preadipocyte differentiation [73]. These data suggest a potential correlation between VDR and adipogenesis. In different phases of adipogenesis, 1,25(OH)2D3 could exert antiadipogenic activity through the WNT/*β*-catenin pathway, the expression of mRNA modulation, and phosphorylation of extracellular regulated kinase via the mitogen-activated protein signaling pathway [80, 85].

Adiponectin is a hormone produced in adipose tissue and the brain. It is involved in the regulation of fatty acid oxidation [86, 87]. Adiponectin is abundant in plasma and is inversely related to body mass index [88]. Therefore, the biological effect of adiponectin can be related to serum concentration. Increased adiponectin in transgenic mice showed that the differentiation of 3T3-F442A cells is reduced through suppression of the expression of preadipocyte factor-1 mRNA and CCAAT enhancer-binding protein [89]. Adiponectin also plays a role in the suppression of metabolic disorders that may cause obesity, nonalcoholic fatty liver disease (NAFLD), or type-2 diabetes mellitus (T2DM) [87, 90, 91]. Moreover, administration of leptin and adiponectin can reverse insulin resistance in mice [92]. 1,25(OH)2D3 treatment can upregulate adiponectin in vitro and inhibit anti-inflammatory cytokine expression, and daily intake of fortified vitamin D can improve inflammation in T2DM [88, 93, 94]. However, data on the effect of 1,25(OH)_2_D_3_ on adiponectin in human adipocytes are lacking. Therefore, active vitamin D probably acts in adipogenesis by affecting insulin resistance, VDR, leptin expression, or inflammatory response [95–100].

## 4. Vitamin D Deficiency with Lipid Metabolism Diseases

### 4.1. Type-2 Diabetes

Diabetes mellitus (DM) is a consequence of metabolic disorders that contributes to the morbidity of obesity [101]. This disease has four types: type-1 DM (T1DM), type-2 DM (T2DM), gestational diabetes, and specific diabetes types with known causes [102]. T1DM, referred to as insulin-dependent DM, is caused by failure to produce enough insulin; T2DM is caused by the inability of the body to respond properly to insulin; gestational diabetes often occurs in pregnant women and could be overcome after pregnancy; and the fourth kind of DM is diabetes with known causes [102, 103]. About 90% of diabetes cases are T2DM. Therefore, insulin resistance is a major factor for T2DM development. The morbidity of T2DM can be affected by environment, obesity, and age [104].

Low concentration of vitamin D is associated with T2DM patients. The serum concentration of 1,25(OH)D in 69.9% of 103 patients is lower than 20 ng/ml and negatively correlated with hemoglobin A1C and insulin resistance [105]. In adults aged over 45 years, vitamin D deficiency is significantly associated with occurrence of T2DM [106]. Substantial evidence shows a link between vitamin D and T2DM [107].

Vitamin D deficiency can inhibit pancreatic insulin secretion; vitamin D can protect *β*-cells through cytokine regulation, promote depolarization by regulating the function of calcium-binding protein on pancreatic *β*-cells, and regulate the concentration of calcium ions and the flow of calcium through the cell membrane [108–111]. Therefore, the potential role of vitamin D is to induce the expression of insulin receptor, promote the expression of PPAR*γ*, or affect glucose transporter activity by regulating intracellular calcium levels [112–114].

Inflammation also participates in contributing insulin resistance. In T2DM patients, 1,25(OH)D can improve insulin resistance through negative regulation of the expression of inflammatory cytokines, such as interleukin-1, interleukin-6, interleukin-8, and tumor necrosis factor *α* [115]. Vitamin D deficiency can affect insulin secretion and resistance; thus, it plays a role in the occurrence and development of T2DM [116–118]. However, these meta-analyses still need to be improved because of the limited concentration used in the study (at least 2000 IU/day) and the short investigation period on the patients.

### 4.2. Nonalcoholic Fatty Liver Disease

NAFLD is a stress-induced liver injury that is associated with insulin resistance and metabolic syndrome [119]. The causes of NAFLD are diabetes, obesity, age, and diet [120]. NAFLD has two types, namely, nonalcoholic fatty liver and nonalcoholic steatohepatitis [121, 122]. This disease is usually treated through weight loss and exercise. NAFLD is the most common chronic liver disorder in western countries [123]. It is a continuous process of liver injury, which may lead to steatohepatitis, cirrhosis, and liver cancer [124]. Vitamin D plays an important role in NAFLD development [125]. About 75% of 5847 insulin resistance and metabolic syndrome patients have vitamin D deficiency [126]. The serum concentration of 1,25(OH)D is lower in patients with NAFLD than in normal patients, and the fatty liver index is negatively correlated with 1,25(OH)D level [127]. Treatment with vitamin D can improve insulin resistance in patients with glucose intolerance [128]. In vivo studies found that vitamin D deficiency and VDR knockdown reduce the secretion of insulin from pancreatic *β*-cells [129]. These results support that low levels of serum 25(OH)D are related to NAFLD.

Vitamin D is associated with insulin resistance phenotypic markers, such as HOMA-IR, ISI, adiponectin, triglyceride, and high-density lipoprotein cholesterol [130, 131]. A prospective study on 524 nondiabetic patients aged 40–69 years has reported that serum 25(OH)D level is negatively correlated with blood glucose level and insulin resistance level [132]. The decrease in insulin sensitivity, pancreatic *β*-cell function, and insulin synthesis and secretion caused by low vitamin D level is related to insulin resistance. Vitamin D deficiency can promote the progress of impaired glucose tolerance, increase the expression of renin angiotensin system components, and damage the transcriptional function of pancreatic genes [133]. Vitamin D also decreases insulin resistance by downregulating the expression of PPAR*γ*2, suppressing the differentiation of 3T3-L1 preadipocytes, and inhibiting adipogenesis [134]. Studies in vivo and in vitro showed that vitamin D is related to the pathogenesis and progress of NAFLD.

### 4.3. Cardiovascular Risk

Previous study indicated that endothelial dysfunction represents an early event in cardiovascular diseases, and there is an association between vitamin D levels and endothelial dysfunction. In addition, vitamin D levels negatively correlated with flow-mediated dilatation (FMD) in many patients affected by type 2 diabetes, whereas current data are still insufficient to confirm vitamin D deficiency or insufficiency lead to an increased cardiovascular risk [135]. The relationship between vitamin D deficiency and cardiovascular risk, as well as mechanism underlying also still needs to be proved by further investigation.

## 5. Conclusions

This review was dedicated to reveal the correlation between vitamin D and adipogenesis, with emphasis on the diseases related to adipose metabolic disorders. Vitamin D has several influences on adipogenesis. Active vitamin D is mainly produced, stored, and degraded in adipose tissue, and VDR is expressed in adipose tissue. Vitamin D affects adipogenesis by regulating the expression of adipocyte transcription factors, such as PPAR*γ*, C/EBP*α*, and LPL, and through affecting insulin resistance, VDR and unliganded VDR, and adipokine secretion.

Adipose metabolic disorders, such as obesity, diabetes, and NAFLD, were specifically chosen in this review. Obesity is a common occurrence worldwide, and it can lead to diabetes and NAFLD. Many studies have indicated that vitamin D deficiency or insufficiency plays an important role in the development and process of obesity, diabetes, and NAFLD. Aside from the diseases discussed in the review, other diseases are also associated with vitamin deficiency, such as hyperlipidemia, ketosis, ketonuria, and atherosclerosis. However, the effect of vitamin D on adipogenesis in lean individuals and the function of calcium in adipogenesis are still keeping elusive. Vitamin D supplements are a promising way to alleviate the burden caused by these diseases. In conclusion, vitamin D administration can provide a new basis for medical therapy.
